# A comprehensive summary of the GISM annual meeting 2025

**DOI:** 10.20517/evcna.2025.61

**Published:** 2025-08-14

**Authors:** Enrico Ragni, Rita Romani, Valentina Grespi, Gabriele Scattini, Giulio Severi, Enrico Lucarelli, Stefano Grolli, Maddalena Mastrogiacomo, Silvia Dotti, Antonietta Rosa Silini, Maurizio Muraca, Filippo Piccinini, Michela Pozzobon, Laura de Girolamo, Ivana Ferrero, Maria Luisa Torre, Augusto Pessina, Luisa Pascucci

**Affiliations:** ^1^Laboratorio di Biotecnologie Applicate all’Ortopedia, IRCCS Ospedale Galeazzi - Sant’Ambrogio, Milano 20157, Italy.; ^2^Department of Medicine and Surgery, University of Perugia, Perugia 06132, Italy.; ^3^Laboratorio Cellule Staminali, Cell Factory e Biobanca, AOSP Santa Maria, Terni 05100, Italy.; ^4^Dipartimento di Medicina Veterinaria, University of Perugia, Perugia 06126, Italy.; ^5^Istituto Zooprofilattico Sperimentale dell’Umbria e delle Marche “Togo Rosati”, Perugia 06126, Italy.; ^6^Osteoncology, Bone and Soft Tissue Sarcomas and Innovative Therapies Unit, IRCCS Istituto Ortopedico Rizzoli, Bologna 40136, Italy.; ^7^Department of Veterinary Medical Science, University of Parma, Parma 43121, Italy.; ^8^Dipartimento di Medicina Interna e Specialità Mediche (DIMI), Università Degli Studi di Genova, IRCCS Ospedale Policlinico San Martino, Genova 16132, Italy.; ^9^Istituto Zooprofilattico Sperimentale della Lombardia e dell’Emilia Romagna, Brescia 25124, Italy.; ^10^Centro di Ricerca “E. Menni”, Fondazione Poliambulanza Istituto Ospedaliero, Brescia 25124, Italy.; ^11^Foundation Institute of Pediatric Research Città della Speranza, Padova 35127, Italy.; ^12^IRCCS Istituto Romagnolo per lo Studio dei Tumori (IRST) “Dino Amadori”, Meldola 47014, Italy.; ^13^Department of Medical and Surgical Sciences (DIMEC), University of Bologna, Bologna 40126, Italy.; ^14^Department of Women’s and Children’s Health, University of Padua, Padova 35128, Italy.; ^15^Stem Cell Transplantation and Cellular Therapy Laboratory, Paediatric Onco-Haematology Division, Regina Margherita Children’s Hospital, City of Health and Science of Turin, Torino 10126, Italy.; ^16^Department of Pharmaceutical Sciences, University of Piemonte Orientale, Novara 28100, Italy.; ^17^PharmaExceed s.r.l., Pavia 27100, Italy.; ^18^CRC StaMeTec, Department of Biomedical, Surgical and Dental Sciences, University of Milan, Milano 20122, Italy.

**Keywords:** Mesenchymal stem/stromal cells, extracellular vesicles, regenerative medicine, nanomedicine, cell therapy, therapeutic priming, clinical translation, drug delivery systems

## Abstract

The GISM Annual Meeting 2025 convened experts in regenerative medicine and nanomedicine to discuss recent advances in mesenchymal stromal/stem cell (MSC) research and extracellular vesicle (EV) technologies. The meeting emphasized novel strategies to enhance the therapeutic potential of MSCs and EVs, addressing both basic biological insights and translational challenges. Discussions highlighted the importance of standardizing production and characterization methods to improve scalability and reproducibility for clinical applications. Emerging therapeutic approaches, including cell engineering and targeted drug delivery, were showcased alongside preclinical and clinical studies. The conference provided a platform for interdisciplinary exchange, fostering collaboration and paving the way toward the clinical integration of EV and cell-based nanomedicine.

## INTRODUCTION

Italian Mesenchymal Stem Cell Group (in Italian, Gruppo Italiano Staminali Mesenchimali, GISM, https://www.gismonline.it/index.php?lang=en) is a leading Italian scientific society dedicated to advancing research and clinical applications of mesenchymal stem/stromal cells (MSCs). It brings together researchers, clinicians, and industry experts to promote knowledge exchange, standardization and innovation in regenerative medicine. Through annual meetings, workshops, and training programs, including initiatives for early-career scientists, GISM supports the development of safe and effective MSC-based therapies and fosters collaboration across academia and industry.

**Figure 1:**
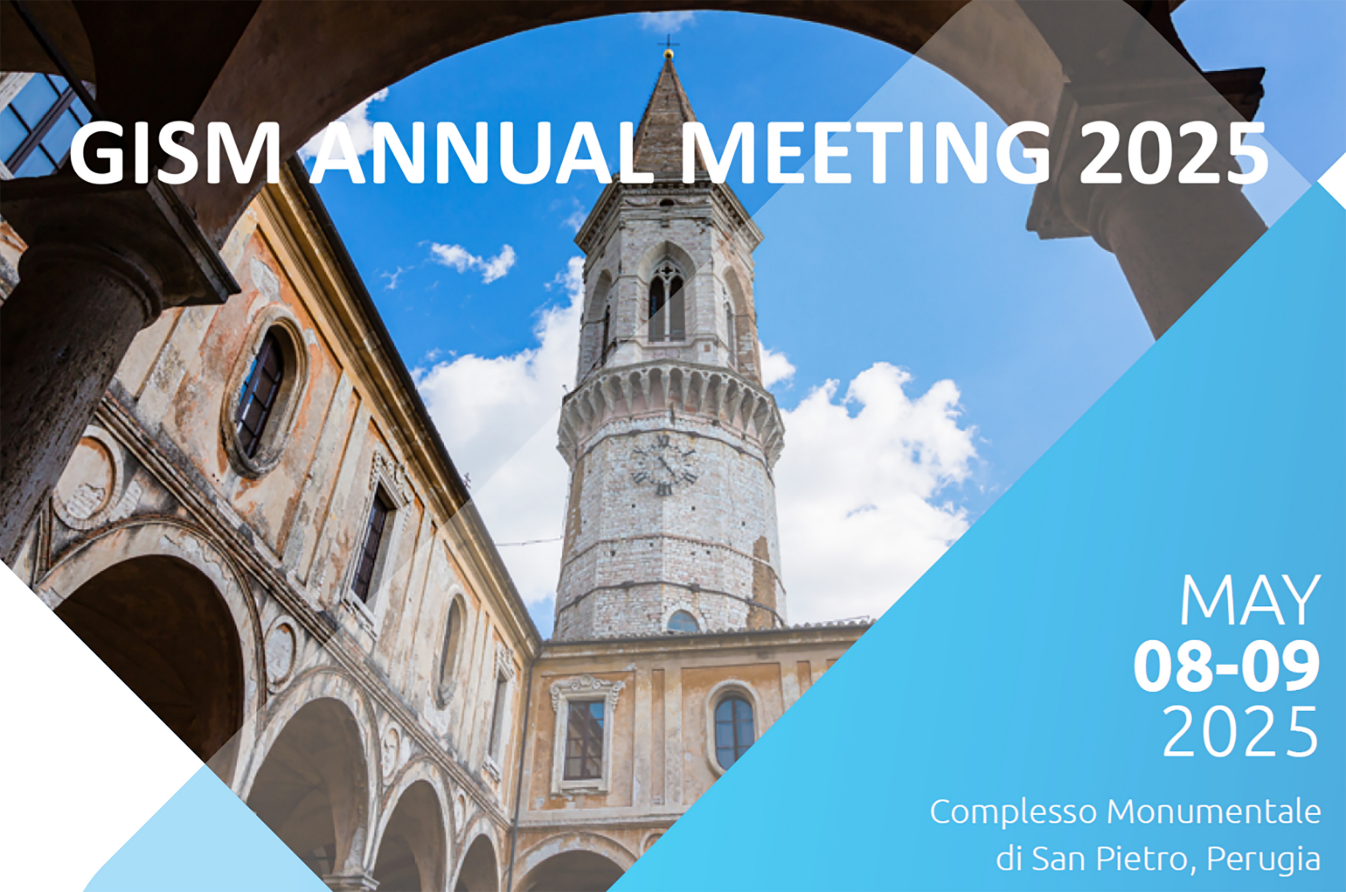
Logo of the GISM Annual Meeting 2025 conference. GISM: Gruppo Italiano Staminali Mesenchimali.

The GISM Annual Meeting 2025 convened in Perugia (Italy) on May 8-9 [Figure 1], reaffirming GISM’s pivotal role in Italy’s MSC research community (https://www.gismonline.it/index.php?option=com_content&view=category&layout=blog&id=72&Itemid=213&lang=en). As a well-established tradition in the national biomedical calendar, the GISM Annual Meeting serves as a dynamic platform for scientists, clinicians, engineers, and stakeholders to share advances in basic research, translational studies, and clinical applications involving MSCs and related cellular therapies.

With a program encompassing emerging technologies like bioprinting and extracellular vesicles (EVs), as well as critical debates on ethics, publishing, and science communication, this year’s meeting drew broad participation across academic generations [Table 1]. Notably, the 2025 edition highlighted the Society’s growing focus on supporting young investigators and critical thinking around research culture. Overall, over 100 participants attended in person, with more than 20 national and international speakers.

**Table 1:** Table summarizing the main preclinical and clinical advances presented

**Session/Presenter**	**Topic**	**Key advances**	**Relevance**
**Day one: summary of preclinical and clinical advances**			
Opening session: how to communicate biomedical science			
Prof. Luca De Fiore (Rome, Italy)	Publishing in medicine: metrics, access, and ethics	Analysis of distorted academic metrics, peer review opacity, and rise of predatory practices	Structural reform in scientific communication
Session 2: GISM next generation			
Prof. Michele Conti (Pavia, Italy)	Bioprinting & engineering for bone regeneration	Dual-head printed PCL-hydrogel scaffolds with mechanical stability and lyosecretome-controlled release	Personalized, bioactive bone implants
Dr. Paola Bisaccia (Padova, Italy)	MSC-EVs in neonatal lung injury	UC-MSC-EVs reduce oxidative stress and fibrosis in rat models of bronchopulmonary dysplasia	Cell-free approach for neonatal therapy
Dr. Pasquale Marrazzo (Bologna, Italy)	Label-Free MSC spheroid characterization	Real-time, non-invasive assessment of MSC spheroids via biophysical measurements	QC innovation in cell therapy manufacturing
Dr. Elia Bari (Novara, Italy)	MSC lyosecretome-enhanced bioprinted scaffold	3D-printed scaffold with controlled release of MSC lyosecretome supporting osteogenic differentiation	Biofunctional bone regeneration platform
Panel discussion			
All	Standardization & translation of MSC-based innovations	Discussion on reproducibility, clinical readiness, and biomaterial integration	Accelerating translation of MSC therapies
**Day two morning: summary of preclinical and clinical advances**			
Session 1: developing MSC-based therapies			
Dr. Almudena Pradera (Madrid, Spain)	Xenogeneic MSC-based treatments	Use of xenogeneic MSCs with immunomodulation strategies to overcome sourcing limitations	Alternative MSC sources for regenerative therapies
Prof. Lia Rimondini (Novara, Italy)	3D *in vitro* models for musculoskeletal diseases	Development of 3D disease models replicating cellular interactions and mechanical environments	Improved preclinical platforms; reduction in animal use
Dr. Giorgio Scita (Milan, Italy)	Biophysical modelling of tumor responses	Application of 2D/3D biophysical models to predict tumor response and optimize MSC therapy design	Enhanced predictive accuracy of preclinical oncology models
Dr. Matteo Longo (Carbonate, Italy)	AMIL link technology in clinical environments	Monitoring and improving disinfection practices to ensure cleanroom compliance in MSC therapy settings	Quality control enhancement for MSC product environments
Session 2: ONE HEALTH			
Dr. Andrew Armitage (Melrose, UK)	MSC therapy in canine osteoarthritis	Standardized outcome evaluation showing MSC efficacy in canine OA models	Veterinary success supporting human OA translation
Prof. Laura Mangiavini (Milan, Italy)	Patient profiling in MSC therapies for knee OA	Stratified approaches using patient characteristics to personalize MSC-based interventions	Precision medicine in regenerative therapy
Prof. Frank Barry (Galway, Ireland)	Clinical reality of MSCs for OA	Critical analysis of translational gaps and the need for rigorous clinical trials	Validation and regulatory awareness for OA therapies
Dr. Michela Taiana (Milan, Italy)	EV microRNA cargo variability by harvesting site	Differences in EV miRNA content based on adipose site and technique; impact on therapeutic performance	Standardization in EV-based MSC therapy
Dr. Kristina Dojchinovska (Perugia, Italy)	Canine MSCs in treating keratoconjunctivitis sicca	Intralacrimal MSC delivery improved tear production and ocular health in dogs with KCS	New cell-based treatment option for dry eye disease
Dr. Mariachiara Stellato (Bologna, Italy)	2.5D analysis of cancer stem cell spheroids	Initial steps in non-destructive extraction of selected cells from spheroids for targeted analysis	Tools for advanced cancer stem cell characterization
**Day two afternoon: summary of preclinical and clinical advances**			
Session 1: from MSCs to extracellular vesicles			
Dr. Daniela Boselli (Milan, Italy)	Purification of MSC-derived EVs	Jet-in-Air cell sorting protocol for high-purity EVs, avoiding ultracentrifugation and enabling subpopulation analysis	Standardized, high-integrity EV production for regenerative applications
Prof. Chiara Gentili (Genova, Italy)	iMSC-EVs for OA therapy	Clinical-grade iMSC-EVs reduced inflammation and oxidative stress in OA models; hydrogel-based delivery	Promising cell-free strategy for OA treatment
Dr. Elena Ceccotti (Torino, Italy)	GMP HLSC-EVs for liver fibrosis	GMP-compliant EVs from liver stem cells improved liver function and reduced fibrosis in murine models	Anti-fibrotic therapy with clinical-grade EVs
Stephane Mazlan (IZON Science, France)	Nanoparticle scalability for clinical use	Scalability and reproducibility solutions for nanoparticle and EV production	Bridging the lab-to-clinic gap for nanoparticle therapies
Marco Lorenzi (Alfatest, Italy)	Advanced EV analysis techniques	Fluorescence-based nanoparticle tracking and single-particle imaging for EV quality control	High-resolution tools for EV research and standardization
Session 2: MSC manipulation/engineering			
Dr. Vitale Miceli (Palermo, Italy)	Priming MSCs to enhance therapeutic output	IFN-γ and hypoxia priming modulate proteomic profile toward immune and angiogenic functions	Customizable MSC-derived products for clinical use
Dr. Francesco Agostini (Aviano, Italy)	GMP bioengineered MSCs for cancer therapy	Human platelet supplements enhanced ASC growth and targeting; improved electroporation for gene delivery	Toward MSC-based targeted anti-cancer platforms
Dr. Lorenzo Andreani (Pisa, Italy)	BMC for aneurysmal bone cysts	Retrospective series showed a high healing rate with BMC injection; average follow-up 33 months	Minimally invasive osteogenic therapy for bone defects
Dr. Paolo Riccardo Camisa (Milano, Italy)	MSC-carried chemotherapy in pancreatic cancer	MSC-nab-paclitaxel targeted liver metastases and modulated immune response in preclinical model	MSC-based drug delivery and immunomodulation in oncology
Dr. Ilenia Motta (Bologna, Italy)	iPSC-MSC models for SDH-deficient GIST	Genome-edited iPSC-derived MSCs modeled SDH-deficient GIST, capturing key molecular pathways	Disease modeling and drug testing for rare cancers

GISM: Gruppo Italiano Staminali Mesenchimali; PCL: polycaprolactone; MSC: mesenchymal stem cell; EV: extracellular vesicle; UC-MSC-EV: umbilical cord mesenchymal stem cell-derived extracellular vesicle; QC: quality control.

## DAY ONE

The first day of the conference featured an incisive opening session on scientific publishing, followed by the GISM Next Generation initiative, a showcase of young researchers contributing to the evolution of MSC-based biomedical innovation.

### Opening session: “Comunicare la Scienza Biomedica” (How to communicate biomedical science)

#### Chair: Augusto Pessina (Milano, Italy)

Following registration and opening remarks, the conference commenced with a thought-provoking keynote by the speaker Prof. **Luca De Fiore** (Rome, Italy), Director of *Il Pensiero Scientifico Editore* (https://pensiero.it/) and representative of the Alessandro Liberati Cochrane Affiliate Center (https://associali.it/). In his talk entitled “On Publishing in Medicine: Impact Factor, Open Access, Peer Review, Predatory Journals and Other Oddities”, De Fiore delivered a compelling keynote that tackled the structural and ethical challenges within modern scientific publishing. With a sharp critical lens, he described the enormous and accelerating output of biomedical literature, now exceeding 2.8 million articles per year, and the disproportionate influence of a few dominant publishers. De Fiore examined the ways in which bibliometric tools like the impact factor and H-index, originally developed to quantify research visibility, have distorted academic incentives, fostering a culture of overproduction and rewarding volume over substance. He highlighted the rise of “mega-authors” who dominate output and questioned the integrity of peer review, which remains an unpaid, opaque and often unrecognized contribution to the scientific ecosystem. Of particular concern was the blurring of lines between established publishers and so-called predatory journals, both of which, he argued, exploit the academic drive to publish while profiting from systemic vulnerabilities. He noted the growing inequalities this system perpetuates, particularly for early-career researchers and women, whose career progression is disproportionately affected by structural publication pressures. De Fiore’s presentation was a call to reevaluate entrenched norms and practices. He advocated for more transparent and equitable models of scientific communication that go beyond metrics to assess real scientific merit, urging institutions to rethink how academic value is defined and rewarded.

### Session 2: GISM next generation

#### Chairs: Filippo Piccinini (Bologna-Meldola, Italy) and Federico Divincenzo (Torino, Italy)

This session was opened by the European Research Council (ERC) Consolidator Grant Winner Prof. **Michele Conti** (Pavia, Italy), University of Pavia and IRCCS Policlinico San Donato, who presented a forward-thinking project that bridges bioprinting technology and engineering design to develop next-generation devices for bone regeneration. His report, “Integrating Bioprinting and Engineering Design to Manufacture Innovative Medical Bioactive Devices”, focused on a novel scaffold system combining structural polycaprolactone with a lyosecretome-enriched alginate hydrogel, co-printed via a dual-head extrusion technique to create composite structures with both mechanical stability and therapeutic functionality. Conti illustrated how computational modeling tools, such as finite element analysis and diffusion simulations, were used to optimize scaffold architecture and bioactive molecule release. The integration of these predictive tools enabled precise control over mechanical properties and lyosecretome delivery, reducing the need for iterative fabrication. Experimental data confirmed the effectiveness of this approach: scaffolds exhibited enhanced stiffness through geometric optimization, and *in vitro* studies demonstrated their ability to support stem cell attachment and osteogenic differentiation, leading to matrix mineralization. By merging digital engineering with regenerative biology, Conti’s work offers a path toward customizable, bioactive implants tailored to both anatomical and biological patient needs. His presentation exemplified the innovative spirit of GISM’s emerging generation of researchers and underscored the translational potential of bioprinting in regenerative medicine.

The afternoon then transitioned to the GISM Next Generation Contest 2024/2025, spotlighting rising talents and their cutting-edge contributions to MSC science and regenerative technologies. In particular, the session focused on the winners of the GISM Next Generation Contest 2024/2025.

- 3rd Place Winner, Dr. **Paola Bisaccia** (Padova, Italy), Università degli Studi di Padova. In her talk entitled “Extracellular vesicles from mesenchymal umbilical cord cells exert protection against oxidative stress and fibrosis in a rat model of bronchopulmonary dysplasia”, she presented preclinical data on EVs derived from umbilical cord MSCs. In a rat model of bronchopulmonary dysplasia, these EVs reduced oxidative stress and fibrosis, demonstrating the promise of cell-free MSC derivatives for neonatal lung injury.

- 2nd Place Winner, Dr. **Pasquale Marrazzo** (Bologna, Italy), Università di Bologna. His talk entitled “Label-free live characterization of mesenchymal stem cell spheroids by biophysical properties measurement” described a novel label-free biophysical characterization method for MSC spheroids, enabling real-time, non-invasive assessment of stem cell cultures. This approach may facilitate quality control in therapeutic manufacturing.

- 1st Place Winner, Dr. **Elia Bari** (Novara, Italy), Università degli Studi del Piemonte Orientale. In his talk entitled “3D bioprinted scaffolds containing mesenchymal stem/stromal lyosecretome: next generation controlled release device for bone regenerative medicine”, he presented a 3D bioprinted scaffold integrated with MSC lyosecretome as a next-generation device for bone regeneration. The work emphasized controlled release and enhanced osteogenic support, aligning with current trends in biofunctional material development.

A concluding panel discussion with the winners underscored key themes, including translational readiness, standardization needs and the future of biomaterials-enhanced MSC therapies. The GISM Next Generation session reaffirmed the Society’s commitment to nurturing early-career researchers and fostering multidisciplinary collaboration.

### Closing

The day concluded with the annual members’ meeting (in Italian, “Assemblea Soci”), where GISM members gathered to review strategic updates and organizational priorities.

## DAY TWO

### Morning

The morning of the second day of the conference focused on two core themes: innovations in MSC product development, predictive modeling, and novel therapeutic indications; and clinical and preclinical advancements in MSC-based therapies for osteoarthritis (OA), with insights from both veterinary and human medicine. The sessions underscored the complexity of MSC-based interventions, the necessity of rigorous characterization and profiling, and the value of translational models in bridging the gap between bench and bedside.

### Session 1: developing MSC-based therapies: from predictive preclinical models to clinical product design

#### Chairs: Katia Mareschi (Turin, Italy), Michela Pozzobon (Padua, Italy) and Marina Torre (Novara, Italy)

The first session transitioned into the translational pipeline, spanning basic science innovations, model development, and novel clinical applications of MSC-based therapies.

Dr. **Almudena Pradera** (Madrid, Spain), Equicord (https://equicord.com/en/), examined the use of xenogeneic MSCs in regenerative therapies with her talk entitled “The Rationale Behind Xenogeneic MSC-Based Treatment”. Her presentation addressed the immunological challenges and potential benefits of using xenogeneic cells, particularly in cases where autologous or allogeneic sources are limited. The study suggested that with appropriate immunomodulation, xenogeneic MSCs could serve as an alternative cell source for treating various conditions, including OA.

Prof. **Lia Rimondini** (Novara, Italy), Università del Piemonte Orientale, delivered a talk entitled “3D *In Vitro* Models for Musculoskeletal Diseases Simulation”, in which she discussed the development of 3D *in vitro* models to simulate musculoskeletal diseases. These models aim to replicate the complex cellular interactions and mechanical environment of musculoskeletal tissues, providing a more accurate platform for testing MSC therapies. The research emphasized the potential of these models in reducing reliance on animal studies and accelerating the translation of regenerative treatments to clinical settings.

Dr. **Giorgio Scita** (Milan, Italy), IFOM Istituto Fondazione di Oncologia Molecolare ETS and University of Milan, explored, with his talk entitled “A Physic Perspective to Unravel Tumour Response Using 2D and 3D Models”, the application of physical sciences in understanding tumor responses through 2D and 3D models. His research highlighted how mechanical forces and cellular architecture influence tumor behavior and treatment responses. By integrating biophysical parameters into preclinical models, the study aimed to improve the predictive accuracy of therapeutic outcomes, thereby enhancing the design of MSC-based interventions in oncology.

Dr. **Matteo Longo** (Carbonate, Italy), AMIL Care Italia SRL (https://www.amil-care.com/), presented in his talk entitled “Monitoring and Control of Disinfection Activities Through AMIL Link Technology: Results of a Multicenter Study and Adoption Strategies” a multicenter study on the implementation of AMIL Link technology for monitoring and controlling disinfection processes in clinical settings. The technology aims to ensure the sterility of environments where MSC-based therapies are prepared and administered. The study reported improved compliance with hygiene protocols and reduced contamination risks, highlighting the importance of stringent quality control in regenerative medicine.

### Session 2: ONE HEALTH - MSCs in OA: what are the results?

#### Chairs: Ana Ivanovska (Galway, Ireland) and Stefano Grolli (Parma, Italy)

The morning session continued with a compelling One Health perspective, focusing on OA as a shared disease burden in both companion animals and humans.

Dr. **Andrew Armitage** (Melrose, UK), Melrose, Roxburghshire, presented a comprehensive analysis of regenerative medicine applications in canine OA entitled “Measuring Treatment Outcomes in Canine OA Treated with Regenerative Medicine”. His study evaluated the efficacy of MSC therapies in improving joint function and reducing pain in dogs. Using standardized outcome measures, including gait analysis and pain scoring, the research demonstrated significant improvements in mobility and quality of life in treated animals. These findings underscore the potential of MSCs as a viable treatment option for OA in veterinary medicine, with implications for translational research in human therapies.

Prof. **Laura Mangiavini** (Milan, Italy), IRCCS Ospedale Galeazzi - Sant’Ambrogio, with her talk entitled “Patient Profiling for Point-of-Care Cell-Based Therapy Products for the Treatment of Knee Osteoarthritis”, discussed the importance of patient profiling in optimizing point-of-care MSC therapies for knee OA. Her research emphasized the role of individualized treatment plans based on patient-specific factors such as serum-protein profiling, age, disease severity, and comorbidities. By tailoring cell-based interventions to the unique characteristics of each patient, the study aimed to enhance therapeutic outcomes and minimize adverse effects. The approach highlights the move towards personalized medicine in regenerative therapies.

Prof. **Frank Barry** (Galway, Ireland), University of Galway, provided a critical evaluation of the current state of cellular therapies for OA with his talk entitled “Cellular Therapy for Osteoarthritis: Reality or Unreality?”. He addressed the challenges in translating preclinical successes to clinical efficacy, including issues related to cell sourcing, delivery methods, and regulatory hurdles. While acknowledging the promise of MSCs in modulating inflammation and promoting tissue repair, he cautioned against overestimating their capabilities without robust clinical evidence. The talk called for rigorous clinical trials to validate the effectiveness of MSC-based treatments in OA.

Dr. **Michela Taiana** (Milan, Italy), IRCCS Ospedale Galeazzi - Sant’Ambrogio, in her talk entitled “Donor Sites and Harvesting Techniques Affect microRNA Cargos of Extracellular Vesicles Released by Human Adipose-Derived Mesenchymal Stromal Cells”, presented findings on how the source and harvesting method of adipose tissue influence the microRNA content of EVs derived from MSCs. The study compared EVs from abdominal and peri-trochanteric fat obtained via surgical excision and lipoaspiration. Results indicated that both the anatomical site and collection technique significantly affect the microRNA profiles of EVs, which in turn may impact their therapeutic efficacy in OA treatments. These insights are crucial for standardizing MSC-derived EV therapies.

Dr. **Kristina Dojchinovska** (Perugia, Italy), University of Perugia, shared findings on the use of canine MSCs in treating keratoconjunctivitis sicca (KCS), a condition characterized by dry eyes due to inadequate tear production. In her talk entitled “Treating Keratoconjunctivitis Sicca with Canine Mesenchymal Stromal Cells”, she described how the study involved intralacrimal transplantation of allogeneic MSCs in dogs with KCS, resulting in significant improvements in tear production and ocular surface health. These results suggest that MSC therapy could offer a long-lasting alternative to conventional immunosuppressive treatments for KCS.

Dr. **Mariachiara Stellato** (Bologna, Italy), University of Bologna, in her talk entitled “2.5D analysis of 3D spheroids for cancer stem cells isolation”, discussed how Cancer stem cells can be studied in controlled environments, such as 3D multicellular spheroids, to better understand their biology and develop treatments that address their drug resistance. She described the first step of a method aimed at extracting individual selected cells without enzymatic digestion. A future step could be to develop a system of micro-pipettes that enables the successful penetration of the spheroid and extraction of selected cells without the need for sectioning.

The session concluded with an engaging discussion on the translational potential of MSC therapies in OA, the need for standardized protocols, and the importance of personalized approaches in treatment planning.

### Poster session

The lunch featured a poster session showcasing a diverse array of research topics, including MSC differentiation pathways, EV characterization, and clinical trial designs for regenerative therapies. Attendees engaged in lively discussions, fostering collaborations and knowledge exchange among researchers and clinicians.

### Afternoon

The two afternoon sessions focused on cutting-edge advances in MSC research and their clinical applications. The first session highlighted innovative techniques for isolating and characterizing MSC-EVs, including the development of clinical-grade EVs for regenerative medicine. The second session showcased translational approaches, featuring clinical case studies on bone regeneration using bone marrow concentrate (BMC) and preclinical research on MSC-based targeted drug delivery. Together, these sessions emphasized the promise of MSC therapies across both orthopedic and oncological fields, underscoring advances in GMP-compliant production and the path toward clinical implementation.

### Session 1: from MSCs to EVs

#### Chairs: Milena Mastrogiacomo (Genova, Italy) and Enrico Ragni (Milano, Italy)

The session focused on strategies to enhance MSC therapeutic potential, as well as optimizing culture conditions to improve MSCs’ efficacy.

Dr. **Daniela Boselli** (Milan, Italy), IRCCS Ospedale San Raffaele, gave a talk entitled “Achieving purity in MSC-derived EVs: a step-by-step sorting protocol”, focusing on novel methodologies to enhance the purification of MSC-EVs, a key tool in regenerative therapies. The team developed a high-sensitivity cell sorting protocol using a Jet-in-Air system with dual-path Forward Scatter optics to isolate CFSE-labelled EVs, avoiding ultracentrifugation that can introduce contaminants and degrade vesicle integrity. The presentation highlighted the preservation of molecular complexity after sorting, supported by microRNA content analysis, and introduced optimized immunophenotyping via spectral flow cytometry to identify novel EV subpopulations. These advancements pave the way for standardized EV-based therapies with improved purity and characterization.

Prof. **Chiara Gentili** (Genova, Italy), University of Genoa and IRCCS Policlinico San Martino, presented, in her talk entitled “Therapeutic application of clinical grade extracellular vesicles secreted by iPSC-derived mesenchymal stromal cells (iMSC)”, preclinical and *in vivo* research on extracellular EVs derived from induced pluripotent stem cell (iPSC)-differentiated mesenchymal stromal cells (iMSCs), exploring their anti-inflammatory and antioxidative properties in OA. The study included the characterization of iMSC lines across passages, clinical-grade EV isolation and phenotyping, and functional assays demonstrating significant downregulation of inflammatory markers and oxidative stress in human chondrocytes. *In vivo* animal models showed that iMSC-EV treatment promotes cartilage regeneration and reduces joint inflammation. The use of a thermosensitive hydrogel for EV delivery was also discussed, which enhances localized and sustained therapeutic effects. These results position iMSC-derived EVs as a promising cell-free approach for OA treatment.

Dr. **Elena Ceccotti** (Torino, Italy), University of Torino, focused her talk entitled “Good manufacturing practice-derived human liver stem cells extracellular vesicles attenuate *in vivo* liver fibrosis” on a translational approach, discussing EVs produced under Good Manufacturing Practice (GMP) conditions from human liver stem cells (HLSCs), a mesenchymal stromal cell-like population with hepatic lineage specificity. The talk covered the scalable production process involving cell stock expansion and EV isolation via Tangential Flow Filtration, followed by formulation and quality control. Using a murine model of thioacetamide-induced liver fibrosis, the study demonstrated that intravenous HLSC-EV treatment reduces fibrosis markers, improves liver histology, and restores liver function. This work highlights the therapeutic potential of GMP-grade HLSC-EVs as anti-fibrotic agents for chronic liver diseases, emphasizing rigorous bioprocessing to ensure clinical applicability.

The translational aim of the session was underscored by two company presentations focused on advanced technologies for EVs analysis and nanoparticle characterization. **Stephane Mazlan** from IZON SCIENCE (https://www.izon.com/), France, discussed the challenges and solutions in achieving scalability and reproducibility of nanoparticle production from laboratory research to clinical applications, highlighting IZON’s approach to bridging this critical gap. Following this, **Marco Lorenzi** from Alfatest (https://www.alfatest.it/), Italy, presented cutting-edge fluorescence-based techniques for sizing and analyzing EVs using Nanoparticle Tracking Analysis and Single Particle Interferometric Reflectance Imaging Sensor technologies, emphasizing their precision and utility in EV research and quality control.

### Session 2: MSC manipulation/engineering

#### Chairs: Ivana Ferrero (Torino, Italy) and Enrico Lucarelli (Bologna, Italy)

The final session showcased cutting-edge research on optimizing MSCs for therapeutic use across regenerative medicine and oncology.

Dr. **Vitale Miceli** (Palermo, Italy), IRCCS ISMETT, presented in his talk entitled “Priming strategies to enhance the therapeutic capabilities of mesenchymal stromal/stem cells: potential implications for their clinical use” a detailed proteomic analysis of conditioned media and exosomes derived from MSCs subjected to two priming strategies: interferon-gamma (IFN-γ) and hypoxia. His team demonstrated that these priming methods induce distinct protein expression profiles in MSC secretomes, with IFN-γ priming enriching proteins related to tissue regeneration and immune responses, while hypoxic priming predominantly enhanced angiogenic factors. These findings highlight the potential to tailor MSC-derived products to improve their clinical efficacy as advanced therapy medicinal products.

Dr. **Francesco Agostini** (Aviano, Italy), IRCCS Aviano, discussed in his talk entitled “Naive or bioengineered mesenchymal stem/stromal cells for potential cancer therapy applications: the importance of culture conditions” the development of GMP-compliant culture protocols for adipose tissue-derived MSCs (ASCs) intended for cancer therapy. Agostini’s group introduced a human platelet-derived supplement that significantly boosts ASC proliferation and enhances their affinity and transmigration toward cancer cells *in vitro*. Additionally, they improved ASC genetic modification efficiency by electroporation without compromising cell viability or homing capabilities. This work paves the way for producing bioengineered MSCs capable of delivering controlled cytotoxic effects against tumors while preserving their intrinsic targeting functions.

Dr. **Lorenzo Andreani** (Pisa, Italy), University of Pisa, shared clinical experience treating aneurysmal bone cysts with autologous BMC. His talk entitled “Treatment of aneurysmal bone cysts with bone marrow concentrate: a case series” described a retrospective series of 46 patients followed for an average of 33 months. The results showed that percutaneous BMC treatment achieved high rates of lesion healing, confirming its use as a safe, readily available, and effective osteogenic therapy for bone defects.

Dr. **Paolo Riccardo Camisa** (Milano, Italy), Vita-Salute San Raffaele University, presented in his talk entitled “Mesenchymal stem cells mediated modulation of the metastatic microenvironment in pancreatic cancer: a step forward clinical translation”, promising preclinical data on using MSCs as carriers for nab-paclitaxel (n-PTX) in metastatic pancreatic ductal adenocarcinoma. His team demonstrated that MSCs loaded with n-PTX selectively homed to liver metastases in mouse models, significantly reducing tumor burden while minimizing systemic toxicity. Importantly, MSC therapy modulated the tumor microenvironment by enhancing anti-tumor immune infiltration, supporting the potential for MSC-based combination immunotherapies in pancreatic cancer.

Dr. **Ilenia Motta** (Bologna, Italy), University of Bologna, described in her talk entitled “SDH-deficient GIST modelling through genome-edited IPSD-derived mesenchymal stem cells” the generation of a novel cellular model for SDH-deficient gastrointestinal stromal tumors (GISTs) using genome-edited iPSCs differentiated into MSC-like cells. This model recapitulates the distinct gene expression signatures of SDH-deficient GISTs, including neural marker upregulation and activation of hypoxia-related pathways, offering a valuable platform for studying disease biology and testing targeted therapies.

The session fostered robust discussions on the future of MSC-based therapies and their clinical translation.

### Poster awards

The poster award session served as an engaging conclusion to the meeting, showcasing a diverse range of high-quality research projects in the field of mesenchymal stem cells and regenerative medicine. Throughout the session, early-career scientists and established researchers presented their latest findings, fostering dynamic scientific exchange and networking opportunities. The evaluation panel carefully reviewed all poster presentations, assessing the scientific rigor, originality, and potential impact of the work. The awards given to Dr. **Valentina Andreoli**, Dr. **Paolo Riccardo Carnisa**, and Dr. **Mariachiara Stellato** recognized outstanding contributions that demonstrated innovation and excellence, highlighting promising directions for future research. This session not only celebrated the achievements of individual researchers but also emphasized the collaborative spirit and advancing momentum within the MSC community. Notably, *EVCNA* (https://www.oaepublish.com/evcna) sponsored the Poster Awards, highlighting its commitment to fostering innovation and excellence within the biomedical community.

### Conclusions

The GISM Annual Meeting 2025 provided a comprehensive platform to advance the understanding of MSC biology and their EV derivatives, emphasizing innovative approaches in priming, engineering and clinical translation. The rich exchange of preclinical and clinical data highlighted the transformative potential of MSC-based therapies and EVs in regenerative medicine and oncology. Moreover, the sessions underscored the critical importance of standardization, scalable manufacturing, and robust characterization techniques to ensure reproducibility and safety in EV and cell-based nanomedicine applications. The collaborative spirit and multidisciplinary insights fostered at the conference pave the way for accelerated development and future clinical implementation of these promising therapeutic strategies.

## DECLARATIONS

### Availability of data and materials

Raw data are available at: https://osf.io/ek34w/?view_only=cfa84f79af8b48b6a9e2542116ec99ae

### Copyright

© The Author(s) 2025.

